# Recycled Sericin Biopolymer in Biotechnology and Bioelectronics

**DOI:** 10.3390/bioengineering12050547

**Published:** 2025-05-20

**Authors:** Davide Vurro, Aris Liboà, Ilenia D’Onofrio, Giuseppe De Giorgio, Zirong Zhou, Vardan Galstyan, Yajie Qin, Xiongchuan Huang, Pasquale D’Angelo, Giuseppe Tarabella

**Affiliations:** 1 Institute of Materials for Electronics and Magnetism (IMEM-CNR), Parco Area delle Scienze 37A, 43124 Parma, Italy; davide.vurro@cnr.it (D.V.); aris.liboa@unipr.it (A.L.); ilenia.donofrio@unipr.it (I.D.); giuseppedegiorgio@cnr.it (G.D.G.); vardan.galstyan@cnr.it (V.G.); giuseppe.tarabella@cnr.it (G.T.); 2 Department of Chemistry Life Sciences and Environmental Sustainability, University of Parma, Parco Area delle Scienze, 43124 Parma, Italy; 3 School of Information Science and Technology, Fudan University, Handan Rd. 220, Shanghai 200433, China; zrzhou24@m.fudan.edu.cn (Z.Z.); yajieqin@fudan.edu.cn (Y.Q.); huangxiongchuan@fudan.edu.cn (X.H.)

**Keywords:** silk sericin, biopolymers, bioelectronics, biotechnology, tissue engineering, biosensors, sustainability, green chemistry

## Abstract

In a world characterized by rapid industrialization and a growing population, plastic or polymeric waste handling has undergone significant transformations. Recycling has become a major strategy where silk sericin has great potential among recyclable polymers. This naturally occurring biopolymer is a sustainable and versatile material with a wide range of potential uses in biotechnology and sensing. Furthermore, preparing and studying new environmentally friendly functional polymers with attractive physicochemical properties can open new opportunities for developing next-generation materials and composites. Herein, we provide an overview of the advances in the research studies of silk sericin as a functional and eco-friendly material, considering its biocompatibility and unique physicochemical properties. The structure of silk sericin and the extraction procedures, considering the influence of preparation methods on its properties, are described. Sericin’s intrinsic properties, including its ability to crosslink with other polymers, its antioxidative capacity, and its biocompatibility, render it a versatile material for multifunctional applications across diverse fields. In biotechnology, the ability to blend sericin with other polymers enables the preparation of materials with varied morphologies, such as films and scaffolds, exhibiting enhanced mechanical strength and anti-inflammatory effects. This combination proves particularly advantageous in tissue engineering and wound healing. Furthermore, the augmentation of mechanical strength, coupled with the incorporation of plasticizers, makes sericin films suitable for the development of epidermal electrodes. Simultaneously, by precisely controlling hydration and permeability, the same material can be tailored for applications in packaging and the food industry. This work highlights the multidisciplinary and multifunctional nature of sericin, emphasizing its broad applicability.

## 1. Introduction

Effective waste management has become one of the challenges of our era. Exponential growth in consumption, coupled with continued dependence on disposable items, has resulted in impressive amounts of waste that exceed the capacity of conventional recycling methods and, consequently, pose severe threats to ecosystems, human health, and the overall well-being of our planet. In this scenario, the concept of the circular economy has emerged as a transformative paradigm, offering a viable alternative to the traditional linear model of consumption [1].

This approach advocates for a closed-loop system founded on three essential principles: (1) waste reduction, (2) reuse, and (3) recycling to minimize waste and conserve resources instead of resorting to end-of-life disposal [2]. This shift is particularly pertinent in the case of polymers, which represent the most abundant source of waste due to their pivotal role in shaping the modern world. Opting for polymer recycling and integrating them into the circular economy emerges as the most advantageous solution to address the challenges associated with waste disposal [3].

Synthetic polymers, particularly plastics, are extensively studied in terms of recycling and the circular economy approach. One of the main synthetic polymers is polyethylene terephthalate (PET), which is widely used in the packaging and textile industries [4]. Since PET is non-biodegradable, reducing its environmental impact involves recycling that requires waste sorting. Mechanical and chemical methods have been employed to give a second life to plastic waste, involving processes such as extrusion (mechanical recycling) [5] and chemical processes [6,7]. However, the recycling of synthetic polymers has disadvantages related to costs and the degradation of their original properties. A modification through the use of additives or fillers aimed at restoring the pristine properties is necessary until the point where modification becomes ineffective and the material is no longer recyclable [8].

Unlike their synthetic counterparts, natural polymers derived from renewable sources, such as cellulose [9,10], starch [11], and proteins [12,13], possess inherent advantages in terms of biodegradability and lower environmental impact. These materials have been utilized for centuries, providing an eco-friendly alternative to synthetic ones.

Among natural polymers, silk, particularly silk sericin (Ser), is currently receiving significant attention in the field of low environmental impact materials and recycling. Ser, which is the outer and gum-like protein of silk threads, is the principal waste of sericulture. For example, China and India, which are the main silk producers globally, had a significant production of 33,770 metric tons in 2020–2021 [14]. Considering that one-eighth of the weight of treated dry cocoons is made of Ser, serious environmental issues may arise from discarding its effluents. Although studies on the toxicologic effects of silk degumming wastewater have shown that Ser does not induce teratogenic effects on living beings in watercourses [15], however, it may cause the eutrophication of water, a process in which oxygen-demanding contaminants decrease the oxygen level in water, leading to increasing algae growth [16]. Hence, Ser recovery within various technological scenarios represents a key factor in addressing environmental issues provoked by its indiscriminate waste.

Sericin, a promising biopolymer, is gaining significant attraction in biomedicine due to its biocompatibility, biodegradability, and ability to enhance cell growth. Ser-based films [17,18,19], sponges [20,21,22], hydrogels [23,24,25], and nanoparticles [26] have been used in drug delivery [27], food packaging [28], tissue engineering [29], cosmetics [30], sensors [31], and textiles [32], and recently, in synapsis-mimicking devices (memristor) [33,34]. However, some recent studies report controversial results on its biocompatibility [35,36]. The extensive attention to Ser’s biotechnology applications suggests that chemical approaches will readily impart such a feature in the coming years. In addition, Ser remains a prospective biomaterial with antioxidant, anti-inflammatory, biodegradability, cell growth, UV protection, and mechanical properties [37]. All these aspects can be tuned and emphasized by modifying the amino acid sequences of Ser and combining them with other materials such as metal nanoparticles or synthetic polymers [38,39,40,41].

In this scenario, this review aims to critically describe the useful aspects of Ser, which is a sustainable material that was, until recently, considered only as a by-product of the silk industry. We discussed its chemical composition, structure, and properties obtained through extraction processes. Afterward, we analyzed all the recent applications of Ser in biomedicine, exploring the field of biosensors for diagnostics and biotechnological applications for therapeutics and health care. Then, we discussed the possible applications of Ser in the food industry.

## 2. Structure and Composition of Silk

Silk is a natural fiber commonly produced by spiders and some insects, including domestic and wild silkworms, moths, honeybees, wasps, and lacewings. Its chemical structure varies depending on the producing organism (i.e., silkworm, spider, and species from Trichoptera and Myriapod [42]). The silk produced by spiders, silkworms, and moths consists of β-sheet crystals embedded in a random coil structure, while the silk from honeybees and wasps is mainly based on α-helices with coiled-coil structures.

Silk structures with high β-sheet content are generally tough, resembling crystallites. The toughness is due to the arrangement of β-layers in the amorphous and hydrophilic phases of the matrix. Therefore, silk can be identified as a water-insoluble biopolymer formed by semi-crystalline fibers.

The most used and studied form of silk is the one obtained from the *Bombyx mori* (*B. mori*) silkworm, a holometabolous insect belonging to the order Lepidoptera and the family *Bombycidae*.

The *B. mori* silk fibers consist of two main proteins, Silk Fibroin (SF, 70–80%) and Ser (20–30%), along with other types of chemical components such as carbohydrates (1.2–1.6%), inorganic matter (0.6–0.7%), wax matter (0.4–0.8%), and pigments (0.2–0.3%) [43,44]. Table 1 summarizes the composition of silk fibers.

Both SF and Ser are in the silk salivary glands of *B. mori*. The silk gland is a typical exocrine gland comprising three areas, i.e., the posterior silk gland (PSG), the middle silk gland (MSG), and the anterior silk gland (ASG).

SF: SF is secreted into the lumen of the posterior glands of the silkworms as a molecular complex that comprises a High (H)-chain 350 kDa, Low (L)-chain 26 kDa, and Glycoprotein P25 30 kDa. The H-chain of SF contains alternating hydrophobic and hydrophilic blocks and provides crystalline-like features to the silk thread compared to the L-chain, which is hydrophilic and relatively elastic. The overall SF complex can maintain its structural integrity thanks to the P25 protein [42].

**Figure 1:**
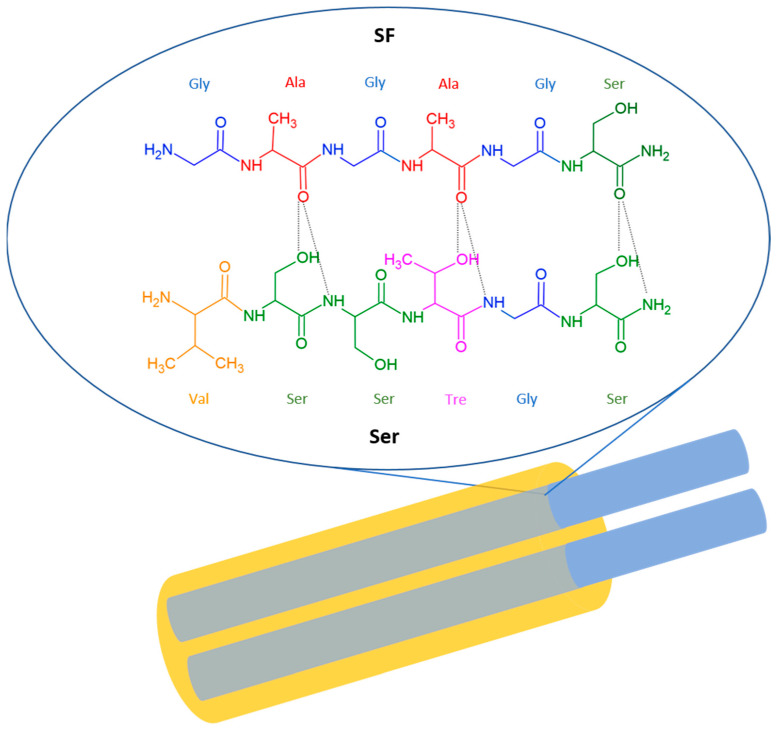
Graphic representation of the hierarchical and chemical structure of silk fiber. Silk is a continuous strand of two SF filaments (shown in blue), coated with an external Ser layer (shown in yellow), which serves as a binder to hold together the SF fibers composing the silk thread. Magnification highlights the intermolecular hydrogen bonding between the silk SF and Ser.

Ser: Ser is secreted by the middle silk gland, which is composed of three secretory vesicle areas, and it contains materials of diverse density and morphology, able to synthesize different types of Ser in the lumen [45]. Figure 1 illustrates the chemical structure of Ser and its intermolecular interaction with SF.

SF has a repeating sequence [GAGAGS]n, while part of the repeating sequence in the Ser has the sequence VSSTGS, with hydroxyl groups regularly spaced on one side of the peptide backbone. The two strands are held together by intermolecular hydrogen bonds formed between the oxygen of CO of SF and the hydrogens of OH and NH of Ser [46].

### Ser Structure and Physicochemical Properties

The fundamental starting point for constructing a clear portrait of the increasingly important role that Ser is beginning to play in the new scientific landscape is its chemical structure. The importance of Ser lies in its properties, which are strictly related to its chemical sequence, as will be described in this section.

Ser is a natural polymer that has the task of enveloping the SF fibers of silk. In the presence of Ser, silk fibers are tough, while after the removal of Ser, they become soft and shiny. This biopolymer is highly hydrophilic and shows a molecular weight ranging from 20 to 400 kDa depending on the extraction methods [45].

There are five distinct types of Ser, numbered from 1 to 5 by notation. The production of these proteins depends on the life cycle stages of *B. mori*. The *Ser1* gene is expressed in the middle (M-MSG) and the posterior (P-MSG) parts of the MSG throughout the silk larvae’s life cycle. Meanwhile, *Ser2* is expressed in the anterior part of the MSG (A-MSG) and encodes two Ser structures (230 kDa and 120 kDa) that act as adhesive molecules. Similarly, *Ser3* is specifically derived in A-MSG and M-MSG. *Ser1* and *Ser3* are major Ser proteins in the cocoon silk, accounting for ~26% and 3% of the total Ser proteins, respectively. The *Ser4* gene was previously identified, showing characteristics like *Ser2* [47].

Recently, Guo et al. reported that *Ser5* is localized in the multilayer structure of Ser and is produced for almost the entire life cycle of *B. mori*. *Ser2*, *Ser4*, and *Ser5* mainly exist in non-cocoon silks. This evidence confirms both a multi-layer spatial structure and varying composition in Ser produced by non-cocoon silk and mature silk [48].

Ser is composed of 18 amino acids, including essential ones, most of which have strong polar side chains (such as hydroxyl, carboxyl, and amino groups) capable of inducing crosslinking, co-polymerizations, and combinations with other polymers [45].

A comprehensive list of amino acids in Ser is reported in Table 2 [49]. The total amount of hydroxy amino acids in Ser is 45.8%, while there are 42.3% of polar amino acids and 12.2% of non-polar amino acid residues [50].

The physicochemical properties of Ser are the result of the relative percentage content of individual amino acids. For instance, the high hydrophilicity of Ser arises from its high content of serine and aspartic acid, approximately 32.16% and 18.71% of content, respectively [49].

This is because the Ser shows a stratified structure where, in this case, the outermost Ser layer (SerA) is the most water-soluble fraction, as it contains the highest proportion of serine and aspartic acid. Conversely, the innermost layer (SerC, in direct contact with SF and separated from Ser by the presence of SerB) is insoluble in hot water. This is due to a gradual increase in non-polar amino acid content, in parallel to the hydrophilic amino acids’ decrease, from SerA to SerC [51].

It is possible to act on the preparation of Ser to control its secondary structure. β-structure Ser is more insoluble than random coil Ser. The randomly coiled structure (63% in partially unfolded Ser) easily transformed to β-sheet structure (set at 35% in unfolded Ser) due to mechanical stretching, temperature, and moisture absorption, causing the sol-gel transition. Hence, solubility occurs when the water temperature exceeds 50–60 °C, while it decreases at lower temperatures, causing the random coil structure to convert into *β*-sheet and resulting in the formation of a gel [52].

Interestingly, Ser is capable of safeguarding cells and tissues from the detrimental effects of reactive oxygen species (ROS) and free radicals. The antioxidant effect of Ser is related to its ROS scavenging ability, anti-tyrosinase properties, and lipid-peroxidation [53,54]. This effect is attributed to the high content of aromatic amino acids, serine, and threonine, whose hydroxyl groups act as chelating centers for active species such as copper and iron [55]. An additional contribution to the antioxidant effect of Ser is provided by pigments such as phenols and flavonoids present in the Ser. These pigments help to reduce damage caused by ROS (i.e., H_2_O_2_), donating electrons to scavenge reactive species. Ser exerts a protective action against UV radiation-induced damage. Acting as a UV barrier, this material can adsorb radiation with wavelengths below 450 nm, thanks to the presence of amino acids such as tyrosine, tryptophan, and phenylalanine [56,57].

Another important ability of Ser is the preservation of biological material during the cryopreservation procedure, in which low temperatures are employed for the storage of cells or biological material (such as semen [58] or ovarian cells [59,60]) for an extended time without altering their functionality. The cryopreservation procedure has several limitations, particularly the tendency to induce oxidative damage to the biological matter, promoting ROS responsible for cell death through apoptosis [61]. ROS are products of various mechanisms, such as osmotic stress and oxidative mechanisms [62,63]. A low concentration of the antioxidant Ser can help to preserve biological materials during the cryopreservation procedure; it can reduce oxidative stress damage, inhibit apoptosis, and maintain the morphology of follicles in the case of ovarian cryopreservation [59].

Ser has gained attention for its potential anti-inflammatory properties and its role in modulating cytokine release. Studies suggest that Ser exhibits anti-inflammatory effects by suppressing the release of pro-inflammatory cytokines, which are signaling molecules involved in the immune response, while increasing anti-inflammatory cytokines. The bioactive compounds in Ser may interfere with the inflammatory cascade, inhibiting the production of cytokines such as tumor necrosis factor-alpha (TNF-alpha) and interleukin-1 (IL-1). These are well-known pro-inflammatory cytokines and are the main ones responsible for the inflammatory reaction cascade [64].

The amino, hydroxyl, and carboxyl functional groups in the Ser backbone are responsible for modifying its chemical and physical properties due to the potential for co-polymerization and crosslinking with other macromolecules. The free amino group of lysine and the hydroxyl group of serine act as suitable binding sites for the polymerization of Ser with other functional polymers, such as glycerol [38,65], polyvinyl alcohol (PVA) [40], chitosan [66], and cellulose [67].

## 3. Ser Extraction Process

The extraction of Ser from silk fiber is a crucial step that can be tailored based on the desired properties and intended applications. Several degumming methods are available for separating Ser from silk fibroin, and the most used techniques will be described in this paragraph, highlighting their effects on the chemical structural composition.

Degumming is a crucial step in the silk manufacturing sector, necessary for improving textile spinning. Through this procedure, it is possible to achieve the separation of the external layers using physical or chemical methods [68]. Physical methods of degumming involve elevated temperatures and pressures or ultrasonic treatments, while chemical methods are performed with various degumming agents. It is important to note that the properties of Ser, such as molecular weight or secondary structure, may be tuned by the method used. For example, when exposed to an alkaline refining process, Ser decomposes to Ser peptides or hydrolyzed sulfur with a molecular weight of less than 20 kDa. In contrast, extraction with acidic solutions allows for the achievement of higher molecular weight fractions that vary from 50 to 150 kDa [69].

All the different approaches used to confer desired mechanical, physical, and chemical properties to Ser (and SF) are schematized in Table 3. The heat-based approach is one of the most implemented due to its cost-effectiveness and simplicity. Meanwhile, the chemical-based approach with sodium carbonate (Na_2_CO_3_) allows for removal of a higher percentage of Ser, but its purification from carbonate requires centrifugation and dialysis processes that are time-consuming [70,71].

To evaluate differences in degumming capability among different approaches, the weight loss determination is a well-known method. It involves comparing the initial weight of the conditioned silk cocoon with the weight after the extraction treatment [72]. Moreover, wettability (water absorbency on degummed samples), tensile strength (measured with a tensile tester), and whiteness are additional indicators of degumming efficiency.

Soap degumming is, together with heat treatment, the most implemented solution and the reference method for all the other proposed ones so far. It consists of using oil-based soaps with a high degree of hydrolysis, such as Marseille soap (MS). The removal of silk gum using a soap solution occurs due to the formation of alkali during soap hydrolysis. This alkali interacts with the outer Ser coating, leading to the synthesis of soda salt. Subsequently, the soda salt dissolves in water due to the emulsifying action of soap. This procedure typically involves temperatures below 100 °C, with a duration varying from 20 to 50 min, and pH adjusted to 9.7 to 10.5. Important aspects for the success of this process include water quality and the low presence of calcium and magnesium ions, which may lead to the formation of insoluble metal complexes with soap [73]. This method has historically been important in the textile industry for imparting luster and softness to silk fibers, enhancing their appearance and dyeability. This technique has often been used in combination with Na_2_CO_3_. A study compares soap degumming performed with MS and in combination with Na_2_CO_3_. Both methods show increased Ser removal with higher concentrations and longer treatment times, achieving similar degumming efficiency under optimized conditions (5 g/L MS or 0.5 g/L Na_2_CO_3_ for 90 min). However, a key finding is that Na_2_CO_3_ treatment retains the color of the self-dyed silk significantly better than MS treatment. Both methods, at the tested concentrations, did not show significant damage to the silk fiber structure. Although widely used, this process results in significant protein degradation, leading to low-molecular-weight peptides. Moreover, it poses purity issues due to residual soap contamination, potential fiber discoloration, and environmental concerns related to wastewater. While effective for the textile industry, its suitability for recovering high-quality Ser, especially for biomedical applications, must be carefully assessed and compared with alternative methods [78,79].

Heating-based procedures can be implemented at ambient and high pressure, using water as a solvent (autoclave method). This technique is often considered more sustainable than chemical methods, as it does not necessarily involve their use and, as a result, avoids residues in the final product and in the effluents. In a 2024 review, a sequential approach is described: boiling in water at 100 °C for 1 h extracts the outer Ser layer (more soluble, ~4.5% of the total mass), followed by autoclaving at 120 °C for 1 h to extract the intermediate layer (~10.5% of the mass). The innermost layer, adjacent to SF, is more resistant and would require chemical agents for complete removal. The outer layer has a higher molecular weight (>30 kDa) compared to the intermediate layer. This method ensures high purity and preserves the bioactivity of Ser, while also providing sterilization due to the high temperatures. However, it requires the use of an autoclave and does not allow for complete recovery of all Ser [75]. Temperature can typically range between 80 and 120 °C with process times from 30 min to 2 h. Pham et al. investigated the optimal conditions to maximize Ser content in solution, which were identified as 120 °C for 30 min, resulting in 37.1 ± 0.72 mg/mL. FTIR analysis confirmed the presence of typical Ser functional groups, demonstrating the maintenance of its biological properties. Finally, concentration-dependent antibacterial activity against *S. aureus* and *E. coli* was investigated, showing a strong antioxidant activity measured by the ABTS assay (up to 88% scavenging at 6 mg/100 mL) [80]. To extract the inner Ser fraction, acids, alkalis, neutral salt solutions, enzymes, or urea can also be employed. Among these, citric acid and Na_2_CO_3_ are the most used. Depending on the abovementioned choice, the properties of Ser change significantly, including thermal stability, molecular weight, and amino acid composition. The duration of the process also impacts the produced Ser, particularly the extraction yield. The extraction yield is 7.7% of the total weight in 30 min at 100 °C. This value increases to 11.4% in 120 min. Increasing pressure and temperature can further enhance these yields; nevertheless, the greater degradation of the extracted Ser must also be considered [81]. An increase in product yield over a longer process also occurs when using chemicals in the extraction process. These substances can also significantly enhance the yields. This was demonstrated by comparing the extraction yields in water with 0.5% Na_2_CO_3_. With extraction times longer than 60 min, sodium carbonate significantly increases the yield.

Ser removal capability varies according to the implemented technique. The secondary structures change based on the extraction methods employed. For autoclaved and urea-degradation samples, no α-helix was present; meanwhile, an abundance of β-sheet was found. On the other hand, Ser obtained through chemical processes like acid-degradation, conventional procedures, and alkali-degradation showed an absence of β-sheet and higher amounts of α-helix [55].

Degumming with Na_2_CO_3_ is the most common and widely used conventional alkaline method. Alkaline conditions and high temperatures promote Ser hydrolysis and increase its water solubility, facilitating its removal from SF [82,83]. Compared to other techniques, it is known for being highly effective, fast (approximately 30 min), cost-efficient, and well standardized [84,85]. However, due to the high pH and temperature, this method causes significant degradation of Ser, leading to a notable reduction in molecular weight (around 15–75 kDa) and alterations in secondary structure due to β-sheet denaturation [77,86]. Prolonged treatments may also adversely affect cytocompatibility. Additionally, concerns have been raised about its environmental impact, as the wastewater generated is highly polluted [87].

Several acids allow degumming in a pH range of 1.5−2 to facilitate a hydrolytic attack on Ser protein, thus breaking peptide bonds between aspartic acid and glutamic acid [73]. Lactic acid, tartaric acid, oxalic acid, citric acid, glacial acetic acid, succinic acid, malonic acid, and sulfuric acid are commonly used acids [88,89,90]. They enable tensile strength improvement, as required by industrial applications [72]. The application of citric acid typically involves immersing silk cocoons in citric acid solutions, usually at 3 wt% [91], using high temperatures or additional techniques like ultrasound. This method is generally considered milder than treatments with strong alkalis. However, some studies suggest that Ser extracted with urea has a higher molecular weight than that obtained with citric acid, indicating significant degradation. This may be due to the fact that, although milder than strong alkalis, acidic conditions, especially when combined with high temperatures, can still result in hydrolysis and degradation of Ser polypeptides [92].

The use of Lithium Bromide (LiBr) as a degumming technique is still rarely discussed in the literature. This method can preserve the structure of Ser almost intact, thereby maintaining its biological functionality. LiBr is a strong chaotropic agent, known for breaking hydrogen bonds and solubilizing structurally robust proteins. However, its action may be overly aggressive or insufficiently selective for controlled removal of only Ser. It could lead to excessive degradation of both Ser and SF, making selective separation difficult or compromising the quality of both components. Additionally, it may be less effective or efficient in selectively solubilizing Ser compared to alkaline or enzymatic treatments. Its main usefulness, therefore, appears to lie in a later stage of the process, ensuring complete solubilization of already extracted Ser [83]. However, for its implementation, Ser is usually subjected to dissolution in a 6M LiBr solution, consistently maintained at a temperature of 35 °C for 24 h. While minor variations exist in subsequent steps, common practices for purification often include adding a 1M Tris-HCl buffer (pH 9.0), followed by dialysis against water using a cellulose membrane with a 3.5 kDa molecular weight cut-off to remove the LiBr. Centrifugation is also typically employed to remove any remaining insoluble material [93,94].

Enzymatic hydrolysis is a relatively unexplored process for short-chain peptide production or Ser hydrolysate [68]. For this purpose, proteolytic enzymes of animal and plant origin are used, such as alcalase, savinase, degummase, papain, and trypsin, which facilitate the hydrolysis of peptide bonds between the carboxyl group of lysine–arginine and the amino group of the subsequent amino acid [43]. Compared to heat-based treatment, this process involves milder conditions of pH and temperature, lower energy, and a shorter duration. It also contributes to hazardous chemical pollution reduction [95]. Microbial and fungal enzymes are reported as deployable for silk degumming. Bacillus species were investigated for their capability to produce alkaline enzymes, and special strains capable of producing Ser-specific protease were isolated and studied in terms of their role in protecting the SF structure against damage [96] due to an overreaction during the process [97]. Enzymatic hydration can yield comparable results in terms of weight loss and absorbency compared to standard washing methods [98]. Fungal strains showed similar satisfactory results on weight loss during the degumming process, enhancing the luster and softness of silk [99,100]. In addition, the introduction of microbial and fungal enzymes paves a way for a large reduction of costs, a notable drawback in enzymatic methods [101].

Aliphatic amines were also proposed as degumming agents due to the presence of a lone pair of electrons on nitrogen atoms, which can potentially act as bases [102]. This allows the hydrolysis of peptide linkages between Ser and SF. Several amines are employed for such applications, e.g., methylamine, ethylamine, dimethylamine, and triethylamine. This method allows for uniform separation regardless of water hardness, but its slowness and unpleasant odor currently restrict its use in industry [73].

Interesting results in terms of efficiency and environmental sustainability have been obtained by implementing supercritical CO_2_. Ser extraction can be enhanced by hydrophilic sites induced by treatment with nonionic surfactants under these specific environmental conditions. A pretreatment with organic acids (i.e., citric or tartaric acid in a pH range of 2–3, 8 h, room temperature) to remove impurities precedes the extraction process, performed in a chamber heated between 95 and 127 °C by maintaining CO_2_ levels between 150 and 400 atm, for a duration of 45 to 70 min [73]. This technology reduces water and energy consumption, avoids microbial contamination, and provides clean Ser. On the other hand, this technology requires significant investments in dedicated equipment.

Physical methods involve high acoustic pressures activated by ultrasonic frequencies, promoting the formation of bubbles in a solution through cavitation. The collapse of the bubbles releases a significant amount of energy that can be exploited to promote the degumming action on silk structures. A comprehensive study on several degumming agents and different operating temperatures shows that ultrasounds at 40 kHz applied for 60 min on silk soaked in warm water (60 °C) achieves the optimal balance between a complete Ser removal and a preservation of SF fiber strength [72]. Long-lasting ultrasonication performed at lower frequencies additionally imparts an increased hydrophilicity and thermal stability, making this technique an attractive solution for the silk industry [103].

The described methods for the separation of silk fibers into Ser and SF exploit various principles and require simple or elaborate processes. Each of these impacts the characteristics of both the as-separated silk components. Ser’s structure and properties can also be modulated accordingly at the production level.

However, some methods explore the production of silk components from silkworms. As mentioned above, the properties of this biological material strongly depend on the isolation process. For this reason, through the implementation of silkworms without posterior silk glands via genetic breeding technology, it is possible to produce Ser filaments. SF-deficient mutant silkworms allow for the extraction of pure Ser using milder methods, such as LiBr, autoclaving, or enzymolysis [104].

Once extracted, Ser is commonly freeze-dried to improve its stability over time, ensuring its availability [83]. Before lyophilization, several other treatments can be implemented. For example, it is possible to ultrafiltrate the solution, allowing the removal of lees and the separation according to molecular weight [76].

As a final remark, Ser/SF production may rely on consolidated physicochemical approaches, as well as on innovative processes that utilize emerging technologies. The implementation of physical methods based on elevated temperatures and pressures is certainly the most commonly used approach for Ser production, as it provides rapid and inexpensive processes taking place in a water environment, also ensuring good yields in case of repeatable processes. Among chemical-based approaches, soap degumming is widely employed for high yields at low costs, allowing for retention of elasticity and resilience of the SF fibers, although Ser remains noticeably degraded. Other chemical-based approaches have gained increasing attention over time because, in different ways, they impart specific features to the final product, such as increased whiteness or reduced hydrolytic action, while lowering costs and permitting the reuse of baths for production on an industrial scale.

While initially more expensive, innovative methods like supercritical CO_2_ extraction and cavitation offer considerable added value by minimizing process waste and maintaining the integrity of the sericin amino acid composition. Supercritical CO_2_ extraction, in particular, offers a solvent-free or minimal-solvent approach, leaving no harmful residues and allowing for selective extraction of sericin based on precise control of its parameters.

## 4. Applications of Ser

The idea that extraction methods may determine the features of silk thread components emerging from the previous section has a significant impact on the various allowable technological applications. The amino acid composition of Ser via choice of the extraction method favors the on-demand improvement of certain properties required by specific technological applications at the material and device level [105,106,107].

A significant example of marketable technologies involves the percentage increase by heat extraction of polyphenols, leading to an increase in antioxidant properties that are essential for applications in cosmetics (i.e., preparation of skin creams) and food packaging. However, the improvement of Ser’s antioxidant properties plays a key role in biomedicine. This property can be used to develop nanosystems acting as carriers that regulate lipid peroxidation and tyrosinase levels for the treatment and prevention of neurodegenerative diseases and cancer [108,109]. Other applicable scenarios in tissue engineering involve the modification of concentrations of methionine and cysteine to enhance cell growth rate, which is useful for scaffold development or wound healing [106,110].

Typically, Ser-dried materials are characterized by low mechanical properties due to extraction processes that affect protein conformation, resulting in reduced resilience because of β-sheet crystallite reduction in favor of a dominant random coil structure [111]. The addition of plasticizers, crosslinked macromolecules, or solvent treatment can overcome this issue, improving mechanical properties that are beneficial for applications in tissue engineering, strain sensors, and wearable devices [32,112]. For instance, the inclusion of PVA enhances the robustness of the Ser film, thereby increasing its tensile properties [112]. However, a high weight ratio between PVA and Ser induces phase separation in the dry-state material. Crosslinking agents, such as glutaraldehyde, have been used to address this problem, resulting in a more uniform film with high mechanical properties (up to a 50% increase in elastic modulus), although with a decrease in elongation. This elongation issue can be mitigated by adding a plasticizer like glycerol [39], which induces an increase in β-sheet content and a decrease in the random coil structure due to hydrogen bonding between Ser and glycerol molecules. In terms of tensile strength, the addition of glycerol enhances elongation to break (from 0.73% to 140%) with only 10% *v*/*v* glycerol content in aqueous Ser [113].

**Figure 2:**
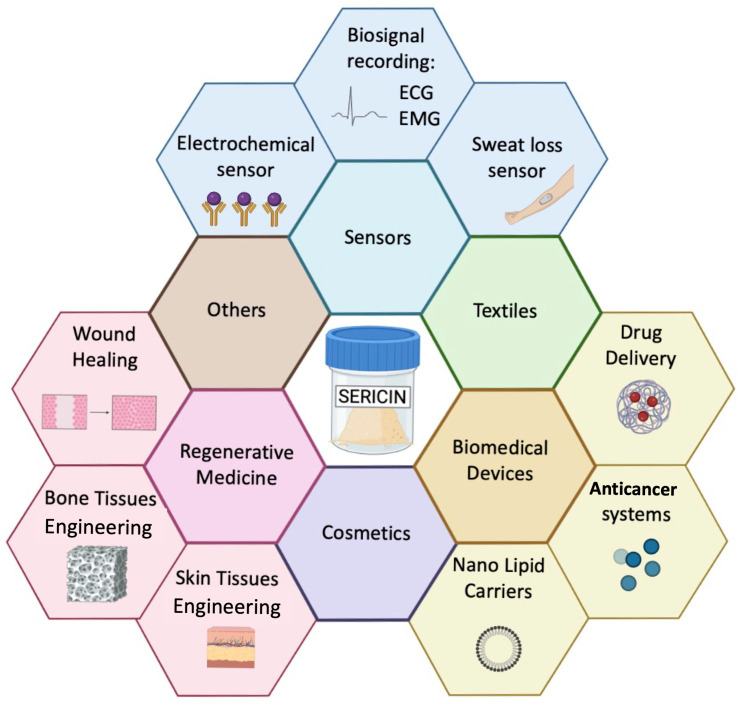
Summary of Ser applications in the biotechnology and bioelectronics fields.

The tunability of mechanical and biological properties represents a valuable benefit for a wide range of applications, particularly in the fields of bioelectronics and biotechnology, as summarized in Figure 2.

### 4.1. Advances in Bioelectronic Devices for Sensing

Electronic/electrochemical sensors are increasingly integrated into everyday objects to implement various functions, including (bio)sensing. In the context of medical diagnostics, the need for constant monitoring the presence of analytes by ASSURED (Affordable, Sensitive, Specific, User-friendly, Rapid/Robust, Equipment-free, and Deliverable) biosensors [114,115,116] is currently pursued by the research community of reference, and flexible sensor platforms for wearable applications are being extensively studied [117,118,119]. The evolution of wearable electronics has, in turn, driven research on biocompatible materials. These materials must seamlessly be interfaced with the human body to ensure the performance of related sensor devices and/or wearable supports (e.g., patches equipped with different sensors) at least to be in line with one of the traditional solutions. In this context, the literature shows how Ser is used as a wearable platform integrated by biosensing systems (electronic textiles) to stabilize bioinks for printed manufacturing and may importantly play an active role in biochemical sensors as a matrix housing recognition elements [108,109] or transducing amplifiers [31,112,120,121].

Electronic textiles are of particular interest, as they combine the functionality of some mature electronic devices currently presented in the literature with the comfort and unobtrusiveness of textiles. Notably, composites and nanostructures can endow electronic textiles with sensing and actuating capabilities, making them highly attractive for healthcare applications [122]. Fabrics can be enhanced with carbonaceous conductive materials like carbon nanotubes (CNTs) or graphene using various deposition techniques, such as screen printing or inkjet printing [123]. However, these materials present challenges due to their high hydrophobicity (water repellency) and instability in aqueous (water-based) environments. To address these issues, stabilizers can be employed to create stable and well-dispersed solutions. Ser, with its amphiphilic properties, can be a useful stabilizer for such solutions, contributing to advancements in electronic textiles [31,121,124,125]. Furthermore, various hydrophilic aromatic groups of Ser allow it to bind with graphene sheets through π-π interactions, stabilizing the dispersion. This property has been used to create stable graphene dispersions for developing breathable, hydrophilic, and washable smart textiles based on polyester fabrics [125]. The proposed Ser/graphene composite enhances the fabric’s wettability, owing to the presence of hydrophilic groups. It has been used to fabricate a smart glove with five sensors positioned on the fingers to record hand motion, creating a multimodal wearable device based on strain sensors and electromyography (EMG) sensors to capture a wider range of complex body movements [125]. In addition, Ser protein can also stabilize carbon black to develop conductive inks. Ma et al. [31] proposed a conductive screen-printable paste based on recycled Ser and carbon black (CB). This paste has been employed in the development of a wearable sensor to monitor sweat loss through a cotton fabric coated with a CB/Ser blend. In this study, the integration of Ser into the sweat fabric sensor enhances the sensitivity to humidity and water content due to its swelling ability. In addition, the introduction of Ser makes the fibers sensitive to pH values. Indeed, the transition from acidic to basic pH causes a change in electrode resistance due to the amphoteric properties of Ser [45,126], and a higher sensitivity is observed in acidic environments due to the amino groups in the Ser structure. Even though normal sweat of a healthy person is usually in the pH range of 4.5 to 7 [127,128,129], this sensor proves to be suitable for accurate sweat detection in real-world environments [31].

CNTs are typically used for electronic textile development. The main constraint for the application of CNTs in textile modification is their high instability in aqueous media, which, in turn, reduces the shelf-life of the modified fibers and the efficiency of fabric modification. Due to its ability to form π-π bonds, Ser efficiently stabilizes CNT solutions, increasing their stability over time (up to one month) [124]. This versatile ink has been utilized in the development of various sensors, including biopotential electrodes, breath sensors, and electrochemical sensors. In the first case, commercial fabrics were dyed with Ser/CNT ink to perform electrocardiogram (ECG) monitoring. These electrodes were compared with commercial ECG electrodes, demonstrating advantages in terms of long-term stability and skin breathability. In the case of breath sensors, the conductive fabric exhibited high sensitivity to breath (air with high humidity) due to the hydroxyl-rich structure of Ser, which is capable of efficiently capturing water molecules. In particular, the absorption of water molecules induced Ser swelling, affecting the electrical resistance of the conductive composite material [124].

In another breath sensor textile application, Ser has been used to stabilize CNT ink in the development of Mxenes/PLA fabrics. Mxenes/PLA fabrics used for breath sensors are characterized by low washing resistance due to the low binding energy between PLA and MXenes due to the scarce presence of hydrophilic groups on the PLA polymeric backbone [130]. The Ser complex endows the desired hydrophilic groups on PLA, while simultaneously increasing the dispersion of Mxenes in water media, thus reducing its oxidative degradation (protective layer effect). This also increases the shelf life and resistance to washing. The addition of CNTs significantly improves the conductivity of PLA/MXenes composite (R_sheet_= 5.5 Ω*sq^−1^), while allowing the textile to maintain its breathability unchanged. The demonstrated application was for humidity measurement with good sensitivity, with the dynamic range of the sensor resistance versus relative humidity calibration curve being from 33% to 97% [121].

Ser’s ability to stabilize carbon materials like CNTs and graphene makes it ideal for developing high-performance electrochemical and flexible sensors to detect hydrogen peroxide (H_2_O_2_) [124]. A printable ink has been developed to fabricate an electrochemical sensor exhibiting a linear dependence on H_2_O_2_ concentration between 0.6 and 17 mM [124]. The use of silver nanoparticles and rGO has been demonstrated to enhance the dynamic range from 0.1 to 10 mM and contextually lower the Limit of Detection (LoD). This sensitivity enhancement is attributed to the high surface area of rGO and to the improved electron-transfer reaction, as induced by the decoration of silver nanoparticles. Here, Ser plays dual roles as a stabilizing agent for CNTs and a reducing agent for graphene oxide and silver [131].

**Figure 3:**
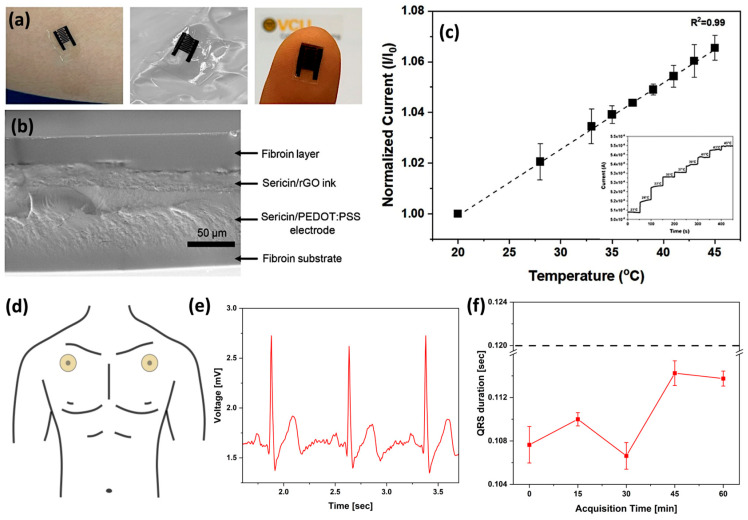
(**a**) Images showing the flexible temperature sensor formed from silk proteins. The sensor may be placed on skin or integrated into a wrinkled surface (e.g., a textile) with a small form factor. (**b**) SEM imaging of the cross-section of the sensor shows the layers that are covalently integrated, preventing delamination and improving stability. (**c**) The calibration curve shows the temperature response of the flexible sensors. The inset shows the signal vs. time measured by chronoamperometry in steps of 5 °C. (n = 3 different sensing experiments). Reprinted with permission from [132]. Copyright 2021 American Chemical Society. (**d**) Schematic of electrode positioning on body. (**e**) Typical ECG waveforms acquired using an SS/PVACaCl_2_ electrode. (**f**) QRS duration as a function of time monitoring of SS/PVA/CaCl_2_ 20 wt% (dotted lines indicate the threshold clinical value of QRS duration for healthy people, i.e., 0.12 s). Adapted with permission from [112]. Copyright 2025 American Chemical Society.

In device manufacturing, Ser modified with 2-isocyanatoethyl methacrylate has also been used as a photoactive material for photolithography. Photo-Ser was added to a rGO suspension in the poly(3,4-ethylenedioxythiophene): polystyrene sulfonate (PEDOT:PSS) conducting polymer to create a bio-ink suitable for photolithographic patterning. An interdigitated electrode has been accordingly developed to fabricate a temperature sensor (Figure 3a–c), achieving high sensitivity in a range between 20 and 50 °C, owing to the phenomenon of temperature-dependent charge transfer behavior [132].

Recently, biodegradable and self-adhesive epidermal electrodes for ECG monitoring were fabricated using a Ser-based film. This film, enhanced with PVA and calcium chloride, allowed for direct skin application and successful ECG recording via a custom system (Figure 3d,e). The resulting QRS amplitudes (Figure 3f) were within the clinically accepted range, demonstrating the viability of this approach [112]. The above examples highlight Ser’s versatility arising from its ability to interact with various conductive materials, like CNTs, graphene, and carbon black, through π-π bonding responsible for the enhanced ink stability. Additionally, Ser possesses both amphoteric and amphiphilic properties. These properties contribute to the development of sensitive breath and sweat sensors, respectively. Table 4 summarizes the role of Ser in wearable and flexible sensors.

From the literature, it emerges that Ser offers easier functionalization, allowing for a customizable range of properties suitable for various stages of fabrication and sensing mechanisms. Ser encompasses within a single material all the functionalities typically found in synthetic polymers that are widely used in sensor applications. Simultaneously, it stands out as a recycled polymer, meaning that its production is environmentally friendly as well. So far, the potential of this material is still underexplored in biosensoristics, considering the richness of functional groups it shows, which is suitable for chemical functionalization with typical recognition elements in biosensors (such as enzymes, antibodies, aptamers, and nanobodies). The use of regenerated Ser can also be envisaged in the context of applications that may potentially benefit from the interfacing between electrolytes and organic conductors [133].

### 4.2. Drug Delivery Applications of Protein-Based Materials

**Figure 4:**
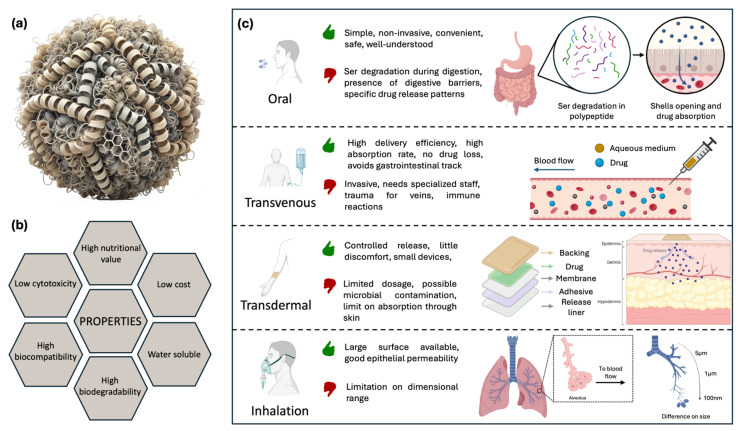
(**a**) Protein-based nanocarrier: schematic diagram of a nanoparticle composed of proteins. The proteins are depicted as folded chains forming a core, with bonds highlighting the β-sheet structure. (**b**) Properties of protein nanocarriers. (**c**) Illustration of major drug delivery routes with advantages, disadvantages, and schematic approach. Oral: Ser degradation by various proteases into peptides leads to the opening of capsules, releasing the drug, which is then absorbed. Intravenous: drug in aqueous solution is released directly into the bloodstream. Transdermal: steady and gradual release of the drug through the epidermis. Inhalation: capsules with reduced diameter can reach the deeper lung regions, enhancing system efficiency.

Currently, protein-based nanocarriers are considered the platform of choice in drug delivery because they offer numerous benefits, such as low cytotoxicity, high biodegradability, and excellent biocompatibility. They also provide high nutritional value, are water-soluble, and cost-effective. These features make them attractive for various applications. Moreover, compared to traditional small-molecule drugs, biological agents offer high activity, high specificity, and minimal nonspecific drug–drug interactions. Therefore, several animal proteins, including keratin, collagen, elastin, and silk-derived proteins, represent a sustainable and affordable alternative to synthetic polymers. These proteins have been extensively studied due to their easy extraction from natural sources and the simplicity of processing under mild conditions. As a result, they exhibit high biocompatibility and possess structural properties that make them suitable for a wide range of biomedical applications. Plant-based proteins like zein, soy protein, and wheat gliadin are some of the proteins used in biomedicine [44]. As it concerns the drug delivery, Ser may be used either in its pure form or as part of integrated matrices, such as scaffolds, hydrogels, foams, capsules, films, fibers, spheres, and microneedles [44]. The popularity of protein-based materials stems from their ability to form β-sheet structures when in a hydrated state [44,134]. Importantly, tuning the β-sheet content in regenerated silk components allows for control of the resilience conferred to micro- and nanocarriers for delivery purposes, as it ensures better stabilization of their structure and preservation of the carrier integrity before reaching the target site. In this respect, it is important to consider modalities of carrier assimilation by the body. Different approaches are schematized in Figure 4a–c.

Oral administration is the most common method, as it is simple, non-invasive, convenient, safe, and well-established, also in terms of nearly complete understanding of various aspects, such as risks, efficiency, and tolerability, achieved so far. While oral administration offers clear advantages, drug delivery can be challenging due to the complexity and specific physicochemical properties of the human gastrointestinal tract that impact drug absorption, particularly for biological agents. These challenges include varying pH levels, cellular and mucus barriers, efflux transporters, and metabolic enzymes [135]. When Ser is ingested, it is partially cleaved by various proteases into peptides, oligopeptides, and amino acids, resulting in sequences of 10–15 kDa. This demonstrates partial digestibility at the gastric level [136]. As mentioned above, to be effective, a drug vehicle must withstand these harsh conditions and only release its content in the intestinal tract. However, for chronic medical conditions requiring controlled release over a prolonged period, oral administration of drug-loaded carriers may not be the optimal solution. Another drawback is that the fast digestion of protein carriers in the gastric tract may induce an excess of drug release when not needed, leading to possible toxicity effects [137].

Beyond oral assimilation, drug delivery can also be implemented through the use of different delivery strategies. The most studied approaches are the transdermal, intravenous, intramuscular, and inhalation routes. Some of these, such as intravenous injections, can increase delivery efficiency because, theoretically, no drug waste occurs during the injection, thus requiring smaller quantities of the drug. On the other hand, drug administration in this case is invasive and requires the presence of a specialized staff for safety purposes [138].

Inhalation represents a valid alternative to oral ingestion. The lungs offer a large surface area, good epithelial permeability, and abundant blood flow, allowing small particles deposited in the lungs to be rapidly and efficiently absorbed into the systemic circulation [139]. A high local deposition rate is ensured by the fact that the particles directly reach the lung surface, bypassing the bloodstream. This allows for high drug concentrations in the target areas, particularly useful for treating pulmonary diseases. This effectiveness is achieved with particles of specific size ranges, as structures smaller than 100 nm can penetrate alveolar tissues, resulting in high absorption rates [140].

Nowadays, transdermal applications are also highly investigated because they allow for controlled release over time with little discomfort, using small and lightweight devices that simultaneously prevent contact with air and potential contamination from bacteria and external agents [141]. By avoiding the gastrointestinal tract, drugs are not subjected to prohibitive pH levels and the presence of digestive enzymes, increasing their bioavailability. Typically, a skin patch is composed of different layers. The backing layer is the outermost, designed to shield the inner layers from external elements. It is generally made from flexible, waterproof materials like polyethylene or polypropylene. The adhesive layer ensures the patch securely adheres to the skin while meeting requirements such as hypoallergenicity. The drug layer contains the active ingredients, formulated to release the drugs steadily over time. The rate-controlling membrane regulates the release rate of the drugs from the patch. Made of semi-permeable materials, it allows the drugs to pass through at a controlled pace. A protective layer covers the patch and adhesive, which should be removed before applying the patch to the skin. This allows the fabrication of user-friendly devices with simple controls, which are now widely used for various purposes in healthcare [142]. However, since not all drugs can be effectively absorbed through the skin, this route may be inefficient and result in the delivery of a limited dosage. Additionally, administration can cause skin irritations, allergic reactions, or microbial growth on the skin [143]. Such devices have been produced using a variety of natural, synthetic, or semi-synthetic materials. Among these, Ser has been implemented in combination with different materials, as thoroughly described in the following sections.

#### Ser in Drug Delivery Applications

Encapsulation represents a considerable and well-studied topic for Ser drug delivery applications due to its chemical reactivity, which allows the easy binding of molecules. In recent years, several researchers have worked to produce drug delivery systems at the micro- and nanoscale, with controlled release patterns through protein-based structures [140,144,145]. The biocompatible Ser is widely implemented for the transport of several compounds for different purposes, from food production to anticancer delivery systems [146,147]. Ser, or its combination with other materials, is often used as a wall material for both hydrophobic and hydrophilic drugs [148]. For instance, a mixture of Ser and maltodextrin (MD) was used to encapsulate anthocyanin from black carrot through a spray drying system. Compared to the control encapsulant, the formulation containing 3% Ser demonstrated not only higher encapsulation efficiency but also better shelf-life under various environmental conditions (4, 25, and 37 °C for 160 days). The latter improvement has been attributed to the increased stability provided by Ser and confirmed by gastric simulation tests. During the gastric stimulation tests, MD/Anthocyanin particles with and without Ser have been exposed to gastric acid for 120 min. A lower release of anthocyanin has been reported for microcapsules based on the Ser/MD mixture due to an increase in the stability of the microcapsule wall. The enhanced stability of the encapsulation has also been demonstrated not to affect the bioavailability of anthocyanins, as tests of controlled release show an increased bioavailability by 7% compared to the control Ser-free sample [149].

Polymer–protein conjugates can also be used to improve the solubility and bioavailability of drugs, extending the circulation time in the body while also reducing the immunogenicity of protein drugs. About 40% of approved drugs and nearly 90% of carrier compounds have poor water solubility [150], making it necessary to develop technologies such as polymeric micelles that can enhance the dispersion and controlled release of insoluble drugs.

**Figure 5:**
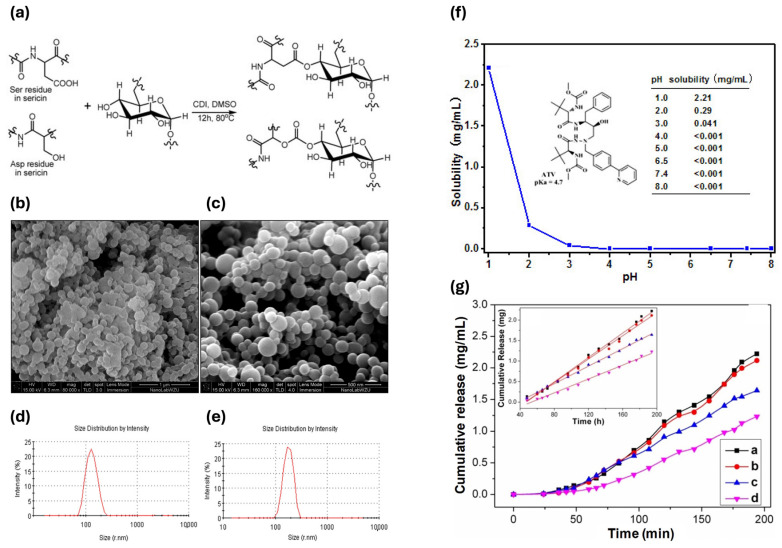
(**a**) Synthesis of Ser/dextran conjugate. (**b**) SEM image of microparticles at the concentration of 5%. (**c**) SEM image of microparticles at the concentration of 10%. (**d**) DLS size distribution of microparticles at the concentration of 5%. (**e**) DLS size distribution of microparticles at the concentration of 10%. (**f**) Native atazanavir dissolution profile. (**g**) Atazanavir-loaded SDC microparticles; inserted image was the linear regression lines (a, pH 2.0; b, pH 6.5; c, pH 7.4; d, pH 8.0) [151].

Ser finds active application in this context. A new Ser/dextran conjugate (SDC) has been developed, which self-assembles into microparticles to improve the solubility of antiviral drugs, such as atazanavir, thereby facilitating controlled release. The conjugation of SDC was carried out using a reprecipitation method (Figure 5a). The optimal concentrations for particle production ranged between 5% and 10%, as shown in Figure 5b,c, and were characterized by a distribution of sizes from 80 to 400 nm (Figure 5d,e). Dissolution experiments of pure atazanavir and atazanavir-loaded SDC microparticles were performed in buffer solutions at different pH levels. The solubility of atazanavir from native drug tablets decreased significantly with increasing pH, starting at 2.21 mg/mL at pH 1.0 and dropping to nearly no release at higher pH values (Figure 5f). In contrast, the SDC microparticles improved atazanavir’s solubility across different pH levels, reaching 2.22 mg/mL at pH 2.0, 1.65 mg/mL at pH 7.4, and 1.24 mg/mL at pH 8.0 (Figure 5g). This enhancement is likely due to the large surface area and the hydrophilic properties of dextran and Ser. The SDC microparticles facilitated a linear release of atazanavir [151].

Another role of Ser in carrier assembly is its use as a second protein coating layer to protect lipid structures of nano lipid carriers (NLCs), which are self-assembling, low-cost, high-loading, and long-term stable nanostructured carriers for drug delivery [152]. The Ser coating improves NLCs’ mucoadhesive properties, promotes cellular uptake, and protects the loaded compounds from enzymatic degradation. As an example, these NLCs are suitable for releasing hesperidin in a controlled manner for the treatment of gastric ulcers, avoiding acute oral toxicity [152]. NLCs also show promise in transdermal drug delivery systems, as they address the limitations of conventional devices. They enhance drug permeation by forming a film over the skin aimed at improving hydration by reducing water loss. Surfactants in NLCs compromise the skin barrier function, further enhancing drug absorption [153].

### 4.3. Ser in Biotechnology Field; Beyond Drug Delivery

Ser’s properties, such as its crosslinking ability (which also provides less cytotoxicity than conventional agents), the control of hydrophobicity/hydrophilicity through its processing, and the improved antioxidant properties and UV protection functions, are the common factors at the basis of allowed applications. Importantly, many applications in biotechnologies may benefit from the feasible combination of such properties in specific compounds and related devices.

In the next sections, an excursus of various applicative scenarios is provided, with a special reference to solutions for the medical field, but also for the food sector. It shows how the direct correlation between material design and processing methods may allow property control, as required by specific applicative objectives. Examples of applications on therapy involving the use of Ser and exploiting its anticancer (Section 4.3.1) and metabolic (Section 4.3.2) effects will be discussed, as well as the potential of Ser in composite formulations for tissue engineering (Section 4.3.3) and the currently relevant approach on wound healing (Section 4.3.4), which will be discussed together with applications impacting the food sector (Section 4.3.5).

#### 4.3.1. Ser’s Anticancer Effect

Anticancer activity exerted by appropriately loaded Ser carriers is one of the most investigated applications in the literature. Folate-conjugated Ser nanoparticles were used for tumor-targeting and pH-responsive subcellular delivery of doxorubicin (DOX). By changing the pH value, these nanoparticles can target the folate-receptor-rich human oral epithelium carcinoma cell line. Indeed, the nanoparticles maintain a negative charge while circulating in the blood flow, which may help minimize non-specific absorption by serum proteins and extend their circulation time. However, once accumulated in the acidic extracellular microenvironment of tumors, their charge excess switches from negative to positive, enhancing cellular uptake due to the strong affinity for negatively charged cell membranes [154].

**Figure 6:**
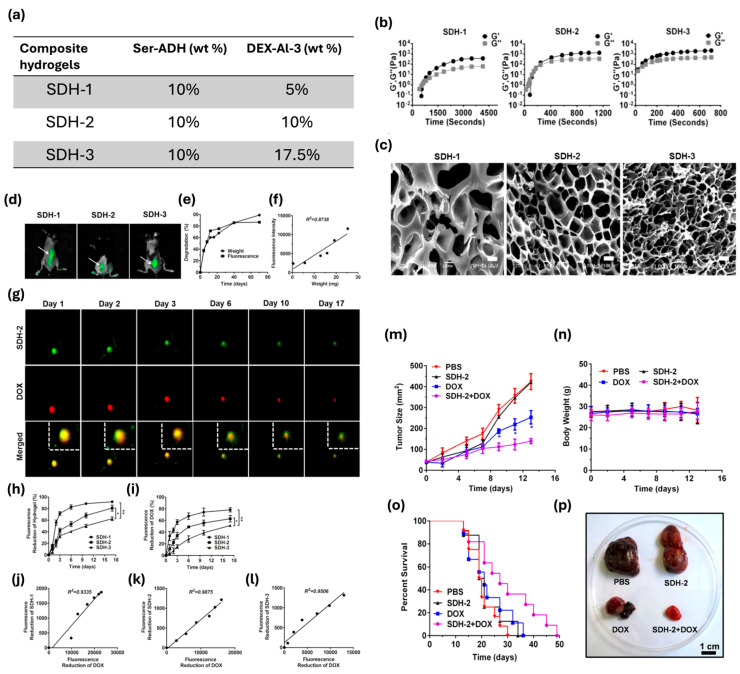
(**a**) Aldehyde content and molecular weight of dextran and derivatives. (**b**) Time evolution of storage modulus (G′) and loss modulus (G″) of SDH-1, SDH-2, and SDH-3 at 15 °C. (**c**) Scanning electron micrographs of SDH-1 (left), SDH-2 (middle), and SDH-3 (right). Scale bars, 10 μm. (**d**) Photoluminescent properties for monitoring hydrogel degradation and drug release in vivo. In vivo fluorescence imaging of C57BL/6 mice subcutaneously injected with SDH-1, SDH-2, and SDH-3 (white arrows) 2 h after injection. (**e**) Quantification of in vivo weight loss and fluorescence intensity reduction of the composite hydrogel (SDH-2) over 70 days. (**f**) Correlation of fluorescence intensity and weight of the SDH-2 hydrogel during in vivo degradation. (**g**) SDH-2 (upper panel) loaded with DOX (middle panel) was injected subcutaneously and degraded over 17 days, monitored by a small animal imaging device using the green fluorescence of SDH-2 (excitation wavelength 420 nm; emission wavelength 530 nm). DOX was observed by its red fluorescence (excitation wavelength 430 nm; emission wavelength 600 nm). The merged images of SDH-2 and DOX are shown in the lower panel. The images outlined by white dotted lines in the upper right corner are enlargements of the merged images. (**h**,**i**) Quantification of fluorescence intensity reduction in (**h**) SDH hydrogels and DOX in vivo at (**i**) the SDH hydrogel sites over 17 days (n = 3 per group per time point; * *p* < 0.05, ** *p* < 0.01; and student’s *t*-tests). (**j**–**l**) Correlation of the fluorescence intensity of DOX with the fluorescence intensity of the (**j**) SDH-1, (**k**) SDH-2, and (**l**) SDH-3 hydrogels during in vivo degradation. (**m**–**o**) In vivo antitumor activities of the DOX-loaded SDH-2 hydrogel. Quantification of (**m**) tumor size, (**n**) body weight, and (**o**) the survival rate in B16–F10-bearing mice receiving PBS, SDH-2, free DOX (DOX), and the DOX-loaded SDH-2 hydrogel (SDH-2 + DOX) [n = 7–12 per group per time point; * *p* < 0.05 (DOX-loaded SDH-2 relative to free DOX); and student’s *t*-tests]. (**p**) Representative image of the isolated tumors on day 14 from the mice receiving the indicated treatments. Adapted with permission from [155], Copyright 2016 American Chemical Society.

Injectable hydrogels also offer a tool for targeted drug delivery in chemotherapy, but in vivo monitoring of drug release and degradation of carriers remains challenging. A hydrazone crosslinked Ser/dextran hydrogel, which is biodegradable and biocompatible, has shown to enable controlled drug release. Hydrogel gelation time and structure can be tuned, changing dextran concentration (Figure 6a–c); the higher the concentration, the lower the gelation time. Also, sericin is a well-known photoluminescent biopolymer that allows a real-time monitoring in vivo test, which may be tightly correlated with the hydrogel weight loss. The proposed hydrogel, when loaded with DOX, significantly has suppressed tumor growth, highlighting its potential to enhance the safety and efficacy of chemotherapy. No changes in body weight have been achieved after DOX treatment, and an increase in death cells was found after 14 days of treatment. Moreover, hydrogels made with Ser show good elasticity, high porosity, and pH-dependent degradation dynamics (Figure 6d–p) [155].

Ser hydrogels can be implanted using minimally invasive approaches, as their physical features are compatible with injectability [156]. For instance, as a potential component of injectable hydrogel for bone regeneration, Ser has demonstrated a low immunological response, inhibiting the host’s foreign body response and reducing inflammation [143].

Versatility of Ser-based hydrogels has been demonstrated for photonic tracing, bio-imaging, electrofluorochromic devices, and chemical and environmental detection [157,158]. Ser hydrogel produced through self-assembly or crosslinking has shown weak self-luminescent properties given by the aromatic residues (tryptophan, tyrosine, and phenylalanine). To improve the fluorescent intensity, Ser hydrogels with a double crosslinking agent can show an improved fluorescent intensity due to the enhanced population of fluorescent groups and a favorable modification of the hydrogel internal structure. Fluorescent hydrogels can be used in the field of biomedicine for monitoring and tracing information, allowing continuous assessment of their biodegradation, biodistribution, and metabolic pathways during in vivo experiments [159].

More recently, the apoptotic effect of Ser extracted in urea was investigated through proteomic and transcriptomic analysis on the viability of HCT116 colon cancer cells. A cell viability assay demonstrated that Ser exhibited cytotoxicity on the cancer cells, with an increase in cytotoxicity correlating with rising Ser concentration and Inhibitory Concentration (IC50) values ranging from 40 to 15,000 μg/mL. Ser induced cell death in 80% of the cultivated cells, as this biopolymer can activate gene expression that stimulates the death receptor pathway, initiating extrinsic apoptosis [160].

#### 4.3.2. Ser’s Metabolic Effect

Metabolic effects on lipid metabolism and obesity can be mediated by Ser via several pathways in the gastrointestinal tract, as well as in the circulatory and immune systems.

**Figure 7:**
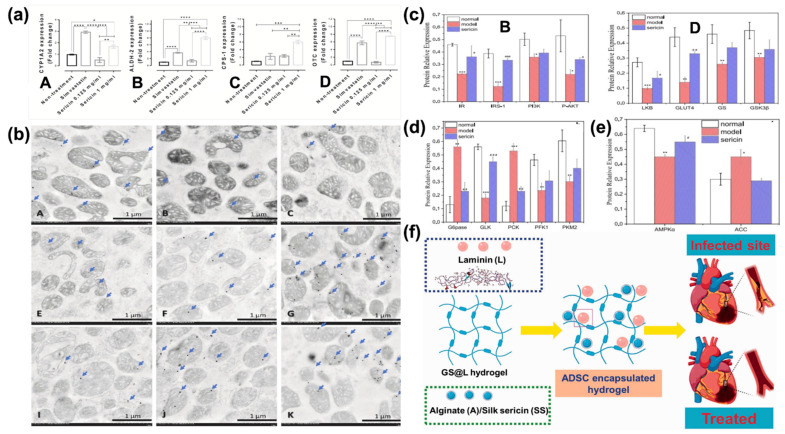
(**a**) The level of cytosol detoxification enzyme and urea cycle enzyme genes in HepG2 cells among treatments. Bar graphs indicated the mRNA fold change expression of CYP1A2 (**A**), ALDH-2 (**B**), CPS-1 (**C**), and OTC (**D**) genes in HepG2-treated with or without simvastatin and two doses of Ser. *; *p* ≤ 0.05, **; *p* ≤ 0.01, ***; *p* ≤ 0.001, ****; *p* ≤ 0.0001. (**b**) CARD-9, MAPK, and LC-3 immunolabelling in the liver mitochondria from the rat among treatments. Electron micrographs show immunogold labelling of CARD-9, MAPK, and LC-3 expressions (arrow) in liver mitochondria extracted from rats without (**A**,**E**,**I**) or with simvastatin (**B**,**F**,**J**) and Ser (**C**,**G**,**K**) treatments. The expression of these markers was located on mitochondrial cristae, matrix, and membrane [161]. (**c**) Effects of Ser on key protein expression of the hepatic insulin signaling pathway in mice. Quantitative analysis of IR, IRS, PI3K, and p-AKT protein expression (**B**) and quantitative analysis of LKB, GSK3b, GS, and GLUT4 protein expression (**D**). * *p* < 0.05, ** *p* < 0.01, and *** *p* < 0.001 versus normal group; # *p* < 0.05, ## *p* < 0.01, and ### *p* < 0.001 versus diabetic model group. (**d**) Effects of Ser on the expression of key proteins of glucose metabolism in mouse liver. Quantitative analysis of G6Pase, GLK, PCK, PFK1, and PKM2 protein expression. ** *p* < 0.01 and *** *p* < 0.001 versus normal group; ## *p* < 0.01 and ### *p* < 0.001 versus diabetic model group. (**e**) Effects of Ser on the expression of key proteins in lipid metabolism in mice. Quantitative analysis of AMPKa and ACC protein expression. * *p* < 0.05 and ** *p* < 0.01 versus normal group; # *p* < 0.05 versus diabetic model group. Reprinted from [162], Copyright 2020, with permission from Elsevier. (**f**) Schematic representation of hydrogel fabrications for myocardial infarctions [163].

Ser can improve hepatic detoxification of nitrogenous compounds, which was studied in an in vitro examination of the urea cycle, a metabolic process occurring in liver mitochondria in which ammonia is converted into urea (Figure 7a,b).

It has been demonstrated that 1 mg/mL of Ser increased the levels of liver detoxification enzymes and urea cycle genes, enhancing hepatic autophagy and accelerating urea synthesis, as indicated by the increased expression of the LC-3 autophagic protein in liver mitochondria. LC-3 is a marker of autophagosome formation. An increase in LC-3 suggests a higher level of autophagy, which, in turn, supports the liver’s ability to manage nitrogen by-products through urea synthesis. Hence, Ser is a promising candidate for the alleviation of hyperammonemia [161]. Although the process requires further investigation, our working hypothesis is that Ser’s antioxidative activity protects mitochondrial cells from oxidative stress. This protective effect may prevent the degradation of healthy mitochondria, resulting in improved urea cycle efficiency.

Ser has also been evaluated for obesity treatment. Integration of the diet with Ser may assist in the decrease in serum lipids, the improvement of glucose tolerance, the enhancement of serum adiponectin, and the restoration of intestinal wall morphometry [30,139,164]. All these functions address a multifunctional character to this biopolymer. Moreover, it has been demonstrated that Ser affects blood cholesterol by lowering its levels. The mechanism of action is mainly exerted at the gastroduodenal level, but there are some assumptions related to a Ser action on cholesterol absorption during the cellular deposition steps [165,166]. Ser assimilation has positive repercussions on other diseases, such as type 2 diabetes and cardiovascular defects. Indeed, Ser regulates standard glucose levels, controls insulin secretion and metabolism, as well as lipid metabolism, and inhibits inflammation. These effects are achieved through enhanced expression of proteins and enzymes, including the insulin receptor, insulin receptor substrate, PI3K, phosphorylated-AKT, hepatic kinase, GLUT4, glycogen synthase, GSK3β, GLK, PFK1, PKM2, and AMPKα (Figure 7c–e). These biomolecules regulate insulin metabolism and glycolysis. Moreover, Ser can modulate the expression of key enzymes related to gluconeogenesis and lipid metabolism in the liver, such as G6Pase, PCK, and ACC. This was confirmed by analyzing type 2 diabetic mice. Here, Ser shows again multifunctional activity, as it significantly decreased fasting blood glucose, fasting plasma insulin, and glycosylated serum protein levels, while also improving oral glucose and insulin tolerances and enhancing antioxidative activities [162]. Regarding cardiovascular defects, injectable hydrogels made of alginate and Ser (Figure 7f) have both shown, upon in vitro and in vivo experiments, the ability to regulate anti-inflammatory factors in cardiomyocytic cells [163].

#### 4.3.3. Ser in Tissue Engineering

**Figure 8:**
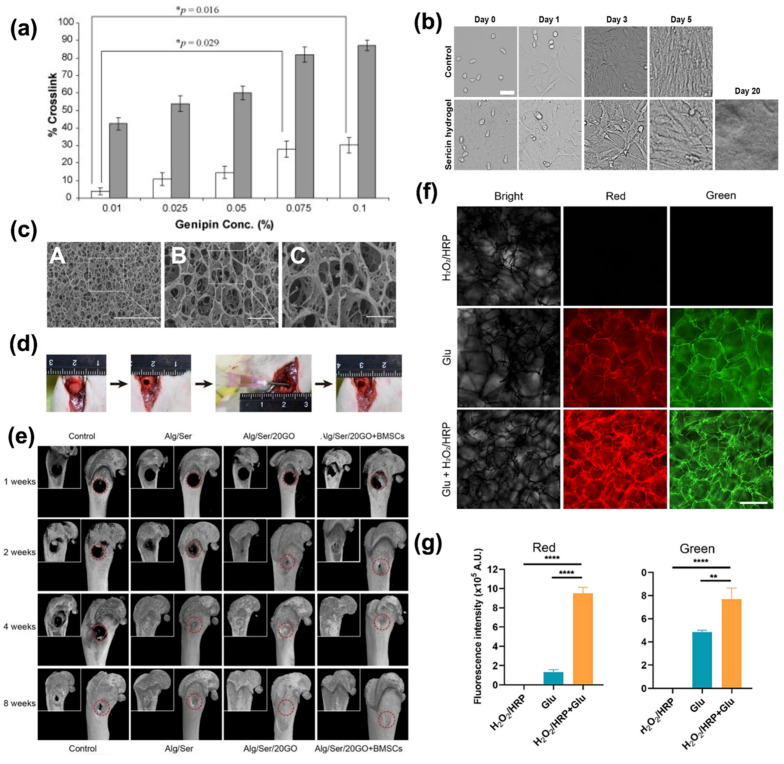
(**a**) Percentage of crosslinks in Ser/PVA/glycerin scaffold with various concentrations of genipin from 0.01 to 0.1% compared with crosslink of the Ser/PVA and Ser/PVA/glycerin scaffolds, respectively. (□) indicates difference in percentage of crosslinks of Ser/PVA/glycerin + genipin with Ser/PVA/glycerin scaffold. (■) indicates the difference in percentage of crosslinks of Ser/PVA/glycerin plus genipin with Ser/PVA scaffold. * indicates significant differences at *p* < 0.05. Reprinted from [169], Copyright 2010, with permission from Elsevier. (**b**) The morphology of mouse myoblast cells (C2C12) growing on the polystyrene surface of the culture dishes (upper panel) and the Ser hydrogel (lower panel) at the different time points after seeding. The cells were initially seeded at the density of 5 × 10^4^ per 35 mm culture dish. Scale bars, 50 μm [156]. (**c**) The cryo-SEM morphology of the Alginate/Ser/GO hydrogel (A-F). Hydrogel implantation into the distal femoral defects and the subsequent micro-CT analysis. (**d**) The procedure of implanting hydrogels into the critical bone defect in rat femurs. The Alginate/Ser/GO hydrogel was injected into the defect with 1.65 wt% alginate, 2 wt% Ser, and 20 μg/mL GO. (**e**) Representative coronal and 3D reconstruction images of micro-CT in rat femurs after 1 to 8 weeks of implantation. Reprinted from [170], with Copyright 2021, with permission from Elsevier. (**f**) The representative images of the hydrogels under the light at the different wavelengths (left column, images are captured under the white light; middle column (red), images (excited at 538–546 nm) are collected using an optical filter with 590 nm; right column (green), images (excited at 480 nm) are collected using an optical filter with 530 nm). Scale bars, 500 mm. (**g**) The quantitative analysis of red and green fluorescence intensity from images. Reprinted from [159], with Copyright 2024, with permission from Elsevier.

Several fabrication techniques, such as emulsification, freeze-drying, porogen leaching, gas foaming, electrospinning, 3D printing, photolithography, and the sol–gel technique [167] can be implemented for the manufacturing of scaffolds for tissue engineering applications. Three-dimensional scaffolds with high porosity provide numerous benefits, as they can serve as a structural support for cell interaction, attachment, proliferation, and, finally, formation of an extracellular matrix. These biodegradable structures allow sufficient transport of gases, nutrients, and regulatory factors while preventing inflammation or toxicity [167]. Ser has been investigated as a natural-based solution to create high-density scaffolds, as it is shown in applications concerning the regeneration of skin and bones, cartilage, and adipose tissues [168,169]. Ser can be coupled with several compounds to form scaffolds with desired characteristics. Of course, this may be allowed by its numerous reactive functional groups. For instance, PVA is employed in Ser blends as a plasticizer, and other substances may affect the morpho-mechanical properties of Ser, allowing, for instance, the tuning of strength and porosity, but also of the stability over time. A Ser-PVA scaffold with 1% *w*/*v* of hydroxyapatite loading was fabricated for bone regeneration purposes, demonstrating an overall improvement in mineral density and suitability in promoting cell proliferation and cytoskeletal organization [168]. Other additives for plasticizing the Ser biopolymer, such as glycerin, demonstrated the capability to reduce phase separation between silk and PVA blends due to the hydrogen bonds between the hydroxyl groups of glycerin and the amide groups of Ser. To increase mechanical properties, such as flexibility, crosslinkers like genipin can be added to the mixture. The addition of genipin to a scaffold based on Ser/PVA and glycerin (Figure 8a) increases the crosslinking percentage, decreasing the compression modulus (from 20 kPa with no genipin to 15 kPa 0.1 wt% genipin). This decrease indicates the formation of the scaffold with high porosity and flexibility [169].

Ser/collagen scaffolds with high porosity, such as to mimic the extracellular matrix, have also been proposed in the literature [171,172]. Chitosan, however, has been blended with Ser to act as a polysaccharide fraction for skin tissue scaffolds, mimicking a skin-like extracellular matrix. The blend can combine the antimicrobial and biocompatibility properties of both composites to promote good cell adhesion of Ser and to achieve a mechanically strong scaffold comparable to the human skin [171].

Injectable hydrogels made of Ser have also shown good elasticity, intrinsic fluorescence (Figure 8f,g) [159], high porosity, pH-dependent degradation dynamics, and fast cell growth (cells grow and survive up to 20 days, as shown in Figure 8b). Hence, Ser has a potential application in injectable hydrogels for bone regeneration thanks to its property of reducing inflammation [156,170]. Ser injectable hydrogel has been proposed for mixing with carbonaceous materials (i.e., graphene oxide) for bone trauma regeneration (Figure 8c–g). The synergic action of Ser and GO facilitates bone regeneration, avoiding surrounding tissue inflammation. Injectable hydrogel based on Ser/GO loaded with BMSC cells induced a complete regeneration of rats’ bone in a few weeks [170].

#### 4.3.4. Wound Healing Applications

Surgeons face significant challenges when dealing with severe acute and chronic wounds, including burns, mechanical trauma, pressure injuries, leg ulcers, congenital skin diseases, and cancer excisions [173]. Burns and skin ulcers lead to significant medical challenges. An ideal wound dressing should keep the wound moist and warm while preventing or minimizing wound infection. Possibly, it must exert an active biological role in tissue regeneration and healing. These goals can be reached whenever wound dressings create a suitable environment for cell adhesion and proliferation, helping restore all the physiological, structural, chemical, and biological skin properties. Inter alia, Ser has been considered as a biomaterial able to mimic the human skin [159], allowing the proliferation of fibroblasts and keratinocytes [139,174]. In recent years, several applications of Ser have been proposed for recovering the healthy physiological state of wounded skin in terms of cell proliferation promotion [175,176,177]. Ser and collagen scaffolds have been suggested to produce stable membranes characterized by easy peeling off, good structural integrity in water, and long-term stability. Highly crosslinked Ser/collagen membranes efficiently absorb moisture, enhancing oxygen permeability and microbial stability [178,179].

**Figure 9:**
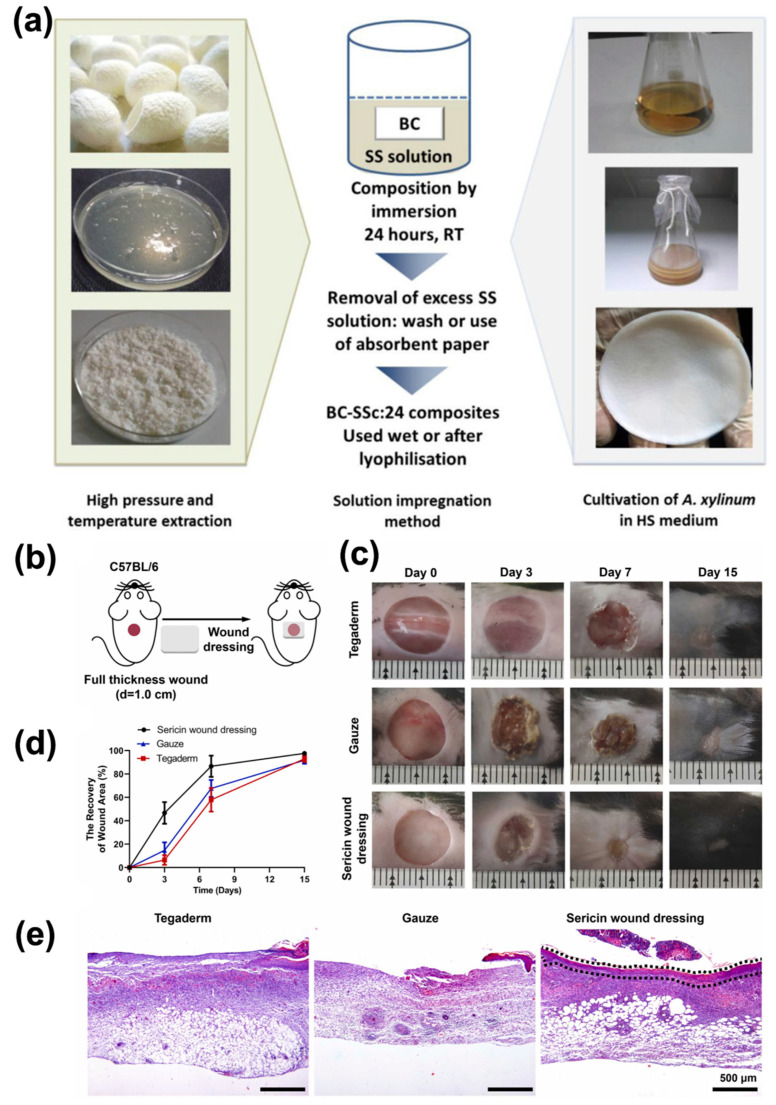
(**a**) Preparation of Ser powder, BC pellicles, and BC/Ser composites. (c = [Ser] in % *w*/*v*, 1, 2, 3; *A. xylinum* = *Acetobacter xylinum*; HS = Hestrin and Schramm). Reprinted with permission from [180], Copyright © 2016, American Chemical Society. (**b**) Ser wound dressing promoted the healing of wounds in mice. Schematic illustration of the treatment procedure for mice acute. (**c**) Representative photographs of the wound closure process were captured in fifteen-day experiments. (**d**) Wound closure rates were evaluated after 3, 7, and 15 days, presented as the percentage of the initial wound area at day 0. (**e**) H&E staining of skin tissues on day 7 after wounding. Scale bar, 500 µm. The black dashed boxes indicate newly formed epithelium. Five mice per group per condition. Reprinted from [181], Copyright 2023, with permission from Elsevier.

However, Ser’s main characteristics constantly face off with poor mechanical properties. Bacterial Cellulose (BC), widely used for wound dressings owing to its excellent mechanical resistance, has been employed to develop BC/Ser composites, which are briefly outlined in Figure 9a. The combination of these two natural biopolymers has been explored in several applications, including skin tissue repair, which is expected to benefit from the nanofibrillar structures of both components [179]. BC/Ser composites are designed to improve wound healing capabilities, allowed by Ser’s cytoprotective and mitogenic effects on mammal cells, whose effect is to promote an enhanced collagen production that, in its turn, accelerates the wound dressing [180].

More recently, the application of unaltered Ser directly produced on a water-absorbent platform from Fib-deficient silkworms was proposed (Figure 9b–e). This Ser typology showed high mechanical properties, excellent stability, and natural biological activities that may be lost during the various processes of silk component extraction. Thanks to its fibrous network structure and 75% porosity, this Ser variant allows excellent air permeability, pH-responsive degradability, softness, high absorbency properties, and, finally, high mechanical strength. High porosity is expected to ensure tissue oxygenation to stimulate epithelialization and fibroblast proliferation and to promote the removal of nutrients and accumulated waste from cells. This material has been tested in vivo in the case of mice for 15 days, showing faster wound closure than regular control gauze and reaching, on day 7, 86.65% of wound closure (20% more than the control) [181].

#### 4.3.5. Ser for Food Application

The production of bioplastics from industrial waste and pollutants is a key goal in creating a sustainable and circular bioeconomy. For this reason, significant efforts are made to develop innovative technologies and solutions in this field. For example, the European Commission’s initiative “New Circular Economy Action Plan (CEAP)”, part of the European Green Deal [182], aims to adopt new strategies for plastic production.

The need to switch from petroleum-based plastic to more sustainable resources is due to several issues (e.g., the increase in micro- and macro-plastics in the environment). Various materials are now employed for packaging production, derived from plants, including polysaccharides and proteins, and microorganisms, such as polylactic acid (PLA), polyhydroxyalkanoates (PHAs), and poly-3-hydroxybutyrate (PHB). These materials are introduced to ensure biodegradability, sustainability, recyclability, renewability, and low-cost production [183,184]. In this context, Ser assumes foremost importance because it can be used to produce bioplastics that are competitive with those currently used in the food industry. The Food and Drug Administration (FDA) has already approved Ser globular protein and its derivatives for inclusion in the generally recognized as safe list (GRAS notice GRN 1026 [185]), with no evidence of causing allergy when administered orally and with no cytotoxicity effects as an ingredient for cosmetics [86].

Moreover, to enhance food quality, active packaging is gaining increasing interest due to its improved functionality in communicating information to consumers. Ser can be used in both food packaging and food formulation, addressing the growing concern over plastic pollution [186]. Due to its weak structural properties and high hydrophilicity, Ser is usually coupled with other biopolymers to be suitable for applications in production processes. These materials can be used for film production in terms of lipid permeability, moisture content, light transmission, swelling, transparency, and solubility [99]. For example, Vijayakumar et al. developed films from bioplastic composites using a formulation that includes Ser, gelatin, and glycerol, suited to food packaging. This formulation demonstrated a tensile strength of 21.03 MPa and 85% biodegradability after 14 days [183]. The findings suggest that Ser could be a viable alternative to traditional plastics, even if transparency and moisture resistance currently limit its application. Other projects have preferentially focused on exploiting the antioxidant and antimicrobial properties of Ser. For instance, films made of Ser and fulvic acid through electrospraying on cellulose were produced and subsequently tested on pear samples, showing an increase in stability over time along a period of 90 days, with good antioxidant activity at lower antimicrobial loads due to the addition of Ser. Antimicrobial activity was tested against *Pseudomonas syringae*, *Botrytis cinerea*, *Penicillium digitatum*, and *Penicillium italicum*. Samples containing Ser exhibited antimicrobial properties, albeit fulvic acid proved more effective in inhibiting cell growth [187]. Although the Ser action may be limited when used alone, these data confirm that it is a perfectly compatible material for the development of new composite formulations with properties that can be applied in the production field.

In recent years, several strategies have been carried out to enhance the mechanical strength of Ser. Among these, blending Ser with cellulose from various sources has been explored, inferring that combining Ser with cellulose nanofibers may affect the tensile properties, improving them [188]. Moreover, the Maillard reaction between Ser and glucose can form a crosslinked film, which has been proposed as an eco-friendly solution for food packaging and drug delivery systems with lower cytotoxicity than conventional crosslinking agents, lower hydrophilicity, and improved antioxidant and UV protection properties [65]. Meerasri et al. proposed to enrich a film made of Ser and pectin with Ser nanoparticles to improve barrier properties and antioxidant activity on food packaging, showing promising results. Also, a reduction in solubility was observed due to the crosslinking between Ser and pectin, enhancing Ser suitability for packaging applications [38].

**Figure 10:**
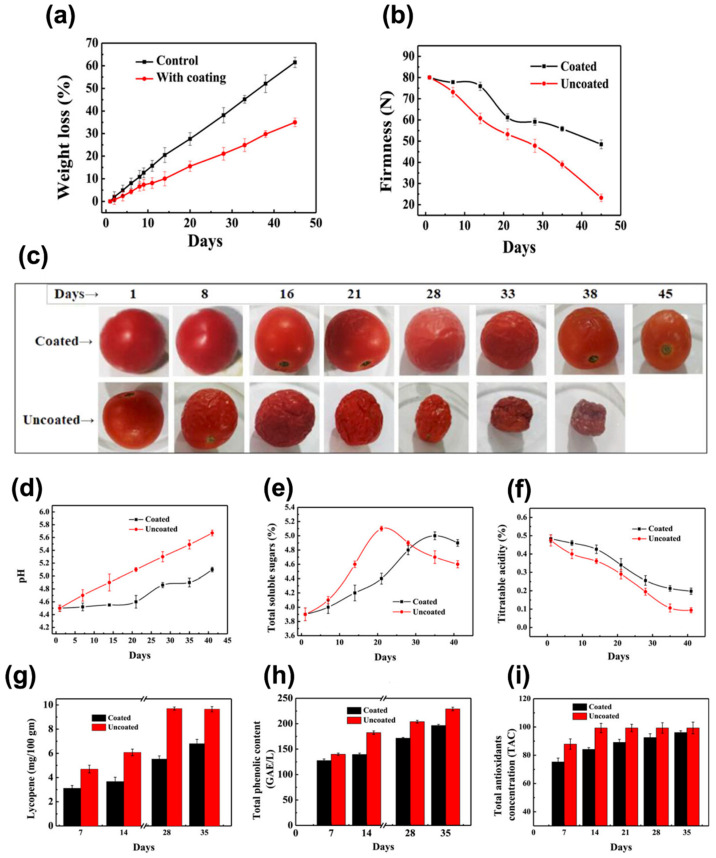
(**a**) Effect of Ser-based edible coating on weight loss. (**b**) Effect of Ser-based edible coating on the firmness for 45 days of storage at 25 °C. (**c**) Photos of the tomatoes with and without the Ser-based edible coating. (**d**) pH. (**e**) Total soluble sugars (TSSs). (**f**) Titratable acidity (TA). (**g**) Lycopene content. (**h**) Total phenolic content (TPC). (**i**) Total antioxidant concentration (TAC) was monitored in tomatoes coated with a Ser-based edible coating at 25 °C. For these experiments, fruits without coating served as control [193].

Currently, regarding food formulation, the implementation of coatings to preserve food quality is carried out due to the need to demonstrate clean labels, with no indication of chemical additives [189]. Silk-based edible coatings can be used to preserve food integrity and quality, as well as to increase the availability of specific target molecules on several foods, like fruits and vegetables, dairy products, meat, poultry, and so on [190]. For example, on fruits and vegetables, these coatings serve to reduce transpiration, respiration, and microbial infections, preserving the physicochemical and phytochemical properties of packaged products [191]. Thanks to the coating presence, water and gas diffusion between foods and the environment is modulated, leading to an increase in shelf life [192]. The coupling of Ser with PVA to produce a hydrophilic coating on bean seeds has shown a reduction of fungal contamination, higher imbibition, and water vapor uptake, providing better germination and reduction of chemicals during storage [18]. Furthermore, other polymers can be implemented to increase the shelf life of vegetable products. Application of Ser, chitosan, aloe vera, and glycerol has been reported to guarantee an increased tomato shelf-life of up to 21 days, without any evidence of wrinkles. Moreover, Ser-based films allow the limitation of weight loss, maintaining high firmness values (Figure 10a–c). At the same time, the presence of the Ser coating positively influences several parameters of food quality, and some of them are listed hereinafter: (i) the juice pH increases in a limited way over time, ensuring the maintenance of optimal values (Figure 10d); (ii) total soluble solids (TSSs) are higher in uncoated products due to cell wall breakdown and subsequent water loss (Figure 10e); (iii) titratable acidity (TA) decreases more slowly for coated samples, showing a delay in ripening (Figure 10f); this is also confirmed by the delayed peak in lycopene concentration (Figure 10g). In addition, total polyphenols, which contribute to color, taste, and aroma, are like untreated samples (Figure 10h), whereas total antioxidant concentration (TAC) increases over time for coated samples instead of remaining constant, suggesting better preservation (Figure 10i). Finally, it was also observed that the presence of the coating reduces surface bacterial contamination [193].

The above-described evidence indicates the potential of Ser-based films in the storage and preservation of fruits and vegetables after harvest. Hence, Ser seems destined to play a key role in addressing significant challenges faced by the food sector, such as spoilage and degradation of food.

Antioxidant properties attributed to its aminoacidic sequence and by the presence of phenolic and flavonoid compounds in the adjacent layers can be considered one of the most important aspects of Ser in determining a positive impact on human health, and this feature explains why its application in the food industry as a natural food preserver increased in the recent past. Conversely, although synthetic antioxidants are more affordable and effective than natural solutions, they are also less appreciated by consumers due to the potential health safety hazard. In this context, a material of natural origin, such as Ser, may find extensive applications within the food industry [194].

Another use of Ser in the food industry involves the low-temperature storage of food. In fact, Ser can act as a cryoprotectant. Cryoprotective proteins can control ice formation inside biological tissues, preventing several species of microorganisms, fish, plants, and insects from proliferating and accidentally being re-crystallized in ice, which is often lethal [195]. Nowadays, this ability is applied in the food industry because it ensures the preservation of the structural properties of frozen foods. Besides the food industry, this capability has also been exploited in other fields, such as tissue engineering and cell culture, where Ser can replace fetal bovine serum in culture media to ensure sustainable costs and environmentally friendly processes. The use of animal-based sera causes serious concerns regarding bovine spongiform encephalopathy. For these reasons, Ser can be considered a valuable substitute for sera-derived products to limit the risk of retroviral infection in implantable materials and during the safe treatment of biological elements [196].

## 5. Conclusions

Ser is a protein extracted from silk that is a versatile and promising biomaterial, with applications spanning biotechnology and electronics. Its inherent properties, such as hydrophilicity and specific amino acid composition, make it a promising material for various applications. The recycling of Ser further enhances its sustainability, offering eco-friendly solutions in materials science. For several years, Ser has been considered just a waste product of industrial silk production and has been converted into effluviant, increasing environmental pollution. Recently, researchers have been re-evaluating silk Ser as a recycled biomaterial to address this issue and fundamentally transform the silk industry and its processing.

The advantages of using recycled Ser in biotechnology are evident in its diverse applications, including drug delivery, tissue engineering, and sensors. The unique properties of proteins, in combination with other polymers, make it possible to obtain materials with specified mechanical and biological characteristics. However, it is crucial to address challenges associated with the recycling process, such as maintaining structural integrity and overcoming phase separation issues.

While the application of Ser has been widely studied in biotechnology, its use in sensor technology is a more recent development, offering exciting new possibilities in just a few works. In the world of electronics, Ser could represent a promising biocompatible material for wearable devices and sensors. Its integration into electronic components can enhance the biocompatibility and sustainability of these technologies. Nowadays, no Ser-based product is on the market in contrast to its counterpart silk Fib, which presents several products from textiles to cosmetics.

The delay in using Ser as a material for commercial products is due to its low immunoaffinity (biocompatibility) with the body and poor mechanical properties resulting from the considerable number of random structures. Several groups have analyzed the biocompatibility issue to demonstrate the immunoaffinity of Ser. Later, the FDA included this material in the list of biocompatible materials. The application of Ser in the sensor development field is in its infancy due to the limitations of Ser-based material mechanical properties. The use of crosslinking and co-polymerization procedures can be adopted to achieve the required properties and the trade-offs between mechanical strength and flexibility. Overall, Ser-based materials remain attractive and promising, combining eco-sustainability with easily tunable biological and mechanical properties. By overcoming the issues, Ser can become an excellent material for manufacturing products and devices, the applications of which will cover a wide range of areas from sensors to innovative medicine.

## Figures and Tables

**Table 1:** Various compositions of silk fibers.

Silk Fibers	SF (70–80%)			Ser (20–30%)
	H Chain	L Chain	P25 Glycoprotein	Glue-Like Protein
Molecular Weight (kDa)	325	26	30	20–400
Polarity	Hydrophobic/hydrophilic	Hydrophilic	Hydrophobic/hydrophilic	Hydrophilic
Structure	Silk I (random coil) Silk II (crystalline structure) Silk II (unstable phase)			No crystalline structure
Function	Core of silk fibers			Wrapped two filaments Coating protein Protection of pupae from UV radiation and external ambient detrimental effects

**Table 2:** List of amino acids of silk Ser.

Amino Acid	Content Percentage
Aspartic acid (Asp)	18.71
Serine (Ser)	32.16
Glutamic acid (Glu)	3.83
Glycine (Gly)	16.43
Histidine (His)	1.46
Arginine (Arg)	3.74
Threonine (Thr)	8.04
Alanine (Ala)	4.35
Proline (Pro)	0.97
Cysteine (Cys)	0.13
Tyrosine (Tyr)	3.14
Valine (Val)	2.56
Methionine (Met)	0.64
Lysine (Lys)	1.79
Isoleucine (Ile)	0.66
Leucine (Leu)	0.80
Phenylalaline (Phe)	0.64

**Table 3:** Ser extraction techniques with their main advantages and limitations.

Extraction Techniques		Peptide Weight	Secondary Structure %				Advantages	Limitations	Ref.
			α-Helix	β-Sheet	Turns	Random Coils			
Chemical-based approach	Soaps	15–75 kDa	28.8	0.0	35.1	36.1	Maximum extraction Fast process Cost-effective High efficiency Strong process Brings silk whiteness High strength and elasticity	High Ser degradation Degummed Ser difficult to recover Energy-consuming Effluent problem Presence of metal ions on soaps can produce insoluble metal soaps on the fiber surface Decrease the fiber strength Degumming bath cannot be repeatedly used	[55,70,72]
	Alkaline solutions	15–75 kDa	28.5	0.0	33.8	37.8	Improved productivity Low processing cost Easy to handle	Impart yellowish color to degummed fibers when used alone	[55]
	Acidic solutions	50–150 kDa	14.9	34.8	17.0	33.3	Improve tensile strength Reusable degumming bath	Dye uptake slightly decreased Limited hydrolytic action	[73]
	Urea	10–225 kDa	2.8	54.5	4.0	38.7	Little SF degradation Cheaper than Marseille soap	Purification is needed to remove impurities Toxic to cells	[74]
	Salt solutions	24–400 kDa	NA	NA	NA	NA	Mild process Low degradation	May cause water pollution Expensive	[75]
Physical processes (heat, pressure)	Boiling	25–150 kDa	NA	NA	NA	NA	No purification process necessary Low cost Environmentally friendly Simple process	Time-consuming SF damaged and Ser degradation Low efficiency Only outer layer removed	[72,75]
	Autoclaving	25–150 kDa	0.0	56.2	2.5	41.3	No purification process necessary Low cost Environmentally friendly High efficiency Avoid contamination	Time-consuming Affects fiber whiteness and absorbency Removes only the outer layer of Ser Incomplete extraction	[76,77]
Enzymatic		5–2 kDa	NA	NA	NA	NA	Avoids uneven dyeing Improved dye affinity (particularly with reactive dyes)	Easy to deactivate High cost Possible overreaction to fibers Time-consuming	[72]
Ammine degumming			NA	NA	NA	NA	Low weighting rate Brings silk bright whiteness Low strength loss	Difficult to apply in industries Unpleasant smell	[73]
CO_2_ supercritical fluid degumming			NA	NA	NA	NA	Keeps Ser clean Avoids contamination	High-cost Necessity of demanding equipment	[72]
Ultrasonication			NA	NA	NA	NA	Less chemical needed Environmentally friendly	Necessity of demanding equipment Fine tuning necessary	[72]

NA = not available.

**Table 4:** Ser’s role in the fabrication of wearable and flexible sensors.

Device	Role of Ser	Sensors	Sensing Mechanism	Output	Ref.
Electronic textile	Carbon black ink stabilizer enhancement of humidity absorption and enhancement of Ph sensitivity	Strain sensor	Change in resistivity	Sweat loss (increasing water volume, good sensitivity in acid media)	[31]
Electronic textile	Graphene stabilizer and chemical site for functionalization	Sweat loss sensor	Electrical mechanism	EMG and hand movement	[125]
Electronic textile	SCNT ink stabilizer	ECG sensor	Electrical mechanism	ECG	[124]
Electronic textile	SCNT ink stabilizer	Breath sensors	Change in resistance	Increase in resistance due to swelling	[124]
Screen-printed electrode	SCNT ink stabilizer	Electrochemical sensor	Amperometric measurement	H_2_O_2_ concentration (linear range from 0.6 to 1.7 mM)	[124]
Electronic textile	Enhancement of MXenes dispersion in water, oxidation inhibitory	Breath sensor	Change in resistance	Humidity level (linear relationship between resistance and RH level from 33% to 97%)	[121]
Electronic textile	SCNT ink stabilizer, reductant of silver ions and graphene oxide	Electrochemical sensor	Amperometric measurement	H_2_O_2_ concentration (linear range from 0.1 to 10 mM)	[131]
Interdigitated electrode	Photoactive matrix to pattern rGO and PEDOT:PSS, biodegradation under proteolysis	Temperature sensor	Linear voltammetry	Linear increase in current in the range from 20 to 50 °C	[132]
Flexible electrode	Ser as skin adhesion layer and electrolyte	ECG sensor	Electrical mechanism	ECG	[112]
